# Association between systemic oxidative stress and visual field damage in open-angle glaucoma

**DOI:** 10.1038/srep25792

**Published:** 2016-05-11

**Authors:** Masaki Tanito, Sachiko Kaidzu, Yasuyuki Takai, Akihiro Ohira

**Affiliations:** 1 Department of Ophthalmology, Shimane University Faculty of Medicine, 89-1 Enya, Izumo, Shimane, 693-8501, Japan; 2 Division of Ophthalmology, Matsue Red Cross Hospital, 200 Horo-machi, Matsue, Shimane, 690-8506, Japan

## Abstract

Local and systemic oxidative stress in intraocular pressure (IOP) elevation and optic nerve damage may be involved in the pathogenesis of glaucoma. We reported previously that a lower level of systemic antioxidative capacity is associated with IOP elevation in open-angle glaucoma (OAG). We assessed the correlation between the visual field sensitivity value, i.e., mean deviation (MD), and systemic levels of prooxidants and antioxidants by analyzing the blood biochemistry in 202 patients with glaucoma. Serum levels of lipid peroxides, ferric-reducing activity, and thiol antioxidant activity were measured using the diacron reactive oxygen metabolite (dROM), biological antioxidant potential (BAP), and sulfhydryl (SH) tests, respectively, using a free-radical analyzer. Univariate and multivariate analyses suggested a positive correlation between MD and BAP (R = 0.005 and *P* = 0.0442 by a multiple regression model adjusted for seven demographic parameters), but no significant associations between the MD and the dROM (R = 0.002 and *P* = 0.8556) and SH tests (R = −0.001 and *P* = 0.8280). Use of more antiglaucoma medication and primary OAG rather than normal tension glaucoma also were associated significantly with worse visual field damage. This large and comprehensive assessment of the association between systemic redox status and visual field damage in OAG suggests that lower systemic antioxidant capacity measured by ferric-reducing activity is associated with more severe visual field damage in OAG that partly explained its roles in IOP elevation.

Glaucoma is a leading cause of irreversible blindness worldwide^1^ including in Japan^2^. The death of retinal ganglion cells (RGCs) and RGC axon loss causes glaucomatous optic neuropathy, in which elevated intraocular pressure (IOP) is the primary risk factor^3^. The IOP in patients with primary open-angle glaucoma (POAG) increases because of reduced aqueous humor outflow at the trabecular meshwork (TM)^4^. Treatment using hydrogen peroxide affects the cytoskeletal structure and cell-matrix interactions in TM cells^5^; depletion of glutathione and hydrogen peroxide treatment decrease the TM outflow facility^6^.

Oxidative stress results from formation of multiple reactive oxygen species including superoxide, hydrogen peroxide, and hydroxyl radicals that can help in the formation and propagation of free radicals. The net oxidative burden between the prooxidant and antioxidant systems is oxidative stress, which damages cellular and tissue macromolecules, resulting in cellular and tissue dysfunction and death. Various oxidative stresses have been reported to induce RGC death in experimental studies^7^,^8^, and free-radical scavengers prevent glaucomatous tissue injury, specifically, glutamate- and IOP-induced RGC death^9^,^10^ and tumor necrosis factor α-induced axonal injury^11^. Evidence suggests that oxidative stress is involved in IOP elevations and RGC loss in POAG and POAG without marked IOP elevation such as that in normal tension glaucoma (NTG).

We reported significantly lower systemic antioxidant capacity levels in patients with OAG including POAG and glaucoma secondary to exfoliation syndrome (EX) compared with controls^12^. Although some studies found a correlation between glaucoma severity (i.e., IOP or visual field damage) and ocular^13^,^14^ or systemic^15^,^16^ levels of oxidative stress in humans, the role of systemic oxidative stress in the pathogenesis of glaucoma is largely unknown. We investigated a possible correlation between visual field damage and systemic levels of prooxidants and antioxidants in OAG (i.e., POAG and NTG).

## Results

We measured the serum levels of systemic oxidative stress markers, i.e., lipid peroxides, ferric-reducing activity, and thiol antioxidant activity using the diacron reactive oxygen metabolite (dROM), biological antioxidant potential (BAP), and sulfhydryl (SH) tests using a free-radical analyzer. Visual field damage was estimated as the mean deviation (MD) value of static perimetry as described in the Methods section. The subject demographic data and MD and oxidative stress marker values in 202 subjects with OAG are shown in Table 1. In this dataset, the average MD, dROM, BAP, and SH test values were −10.5 ± 10.1 decibels, 354.6 ± 62.8 U.Carr, 1,955.8 ± 276.7 μmol/L, and 612.9 ± 98.1 μmol/L, respectively.

By univariate analyses (Table 2), a lower BAP value was associated significantly (r = 0.1550 and *P* = 0.0276) with worse MD, while the dROM (r = −0.0121 and *P* = 0.8644) and SH test (r = −0.0380 and *P* = 0.5918) results were not associated with MD. Other than the BAP, other parameters possibly associated with worse MD were use of a higher number of glaucoma medications (*P* < 0.0001), an IOP higher than that previously recorded (*P* = 0.0005), and higher diastolic blood pressure (DBP) (*P* = 0.0068) among the continuous parameters (Table 2), and among the categorical parameters male gender (*P* = 0.0018), POAG rather than NTG (*P* = 0.0001), and current smoking (*P* = 0.0198) (Table 3).

To adjust for the possible confounding effects by other parameters, the association between MD and systemic oxidative stress markers were analyzed by multiple regression analysis. Seven parameters that showed a significant association with the MD by the univariate analyses were substituted into the multiple regression model together with one of the oxidative stress markers in each model (Table 4). The positive correlation between the MD and BAP remained significant (R = 0.005 and *P* = 0.0442) after adjustment for seven demographic parameters. Multivariate analyses did not find an association between the MD and dROM (R = 0.002 and *P* = 0.8556) or the SH test (R = −0.001 and *P* = 0.8280) (Table 4).

## Discussion

The current study, which included 202 subjects with OAG, is the largest such study to assess the systemic redox status in glaucoma^15^,^17^,^18^,^19^,^20^,^21^,^22^,^23^,^24^,^25^,^26^,^27^,^28^,^29^. Yagci *et al*. reported increased protein carbonylation, a measure of protein oxidation, in aqueous and serum samples from patients with EX compared with controls^19^. Koliakos *et al*. identified significantly lower levels of antioxidative stress enzyme catalase activities in aqueous and serum samples from patients with EX compared with controls^20^. Nucci *et al*. also reported significantly lower total antioxidant capacity in aqueous humor and blood samples from patients with POAG compared with controls^26^. These studies indicated that the systemic antioxidant capacity reflects the local ocular redox status.

A few studies have evaluated the association between systemic oxidative stress and visual field damage. A significant correlation was found between a higher aqueous humor 8-hydroxy-2′-deoxyguanosine (8-OHdG) level, a marker of oxidative stress-induced DNA damage, and a higher 8-OHdG level and lower antioxidant capacity levels in serum samples from patients with glaucoma (n = 28) but not between local/systemic 8-OHdG levels and the visual field MD^15^. A significant association was reported between the systemic vitamin E level and the clinical glaucoma parameters (n = 160), that is, IOP, optic nerve head alterations, and visual field sensitivity; however, the study included mixed types of glaucoma in a case group^23^. The various types of glaucoma, i.e., open-angle and angle-closure glaucomas or primary and secondary glaucomas, have different etiologies; thus, separate analyses of each glaucoma type seemed more appropriate. Current study is the largest and the most comprehensive assessment of the association between systemic redox status and visual field damage in POAG, and the significant association identified between the MD and BAP in the current study was unique. The difference in the sample number may explain the discrepancy between the previous and current studies.

A few groups have investigated the effects of local or systemic oxidative stress on glaucoma parameters. Increased 8-OHdG levels in TM specimens from humans were associated with higher IOP^30^ and more severe visual field loss^13^,^14^ in OAG, and increased aqueous humor oxidative stress was associated with higher IOP in patients with EX^31^. We found previously that the systemic BAP level was lower in patients with higher IOP levels. The odd ratios suggested that, compared with subjects with the highest BAP value, subjects with the lowest BAP value have, respectively, 17 and 25 times higher chances of being classified into the IOP groups with the second highest and highest values^16^. The current study showed that, other than the BAP, use of more glaucoma medications and the presence of POAG rather than NTG were associated consistently with more severe visual field damage (Table 4). Considering these findings, in addition to the direct insults by oxidative stress on the RGCs, decreased systemic antioxidant capacity may be associated with neuronal damage through elevated IOP.

We did not identify a significant association between the dROM level and MD; this agreed with our previous observation of no significant association between the dROM and IOP levels^16^. Glaucomatous damage in the TM or neuronal cells may result from local increases in oxidative stress due to local compensation for systemic oxidative stress by systemic reduction of antioxidant capacity in each individual. The current study also found no significant association between the SH and MD. The glutathione and thioredoxin systems may be major thiol-mediated redox systems in humans, although the plasma glutathione level is 100 to 1,000 times higher than that of thioredoxin^32^,^33^. A negative correlation was found between age and total glutathione levels in red blood cells with no significant difference in the total glutathione level between patients with glaucoma and controls^18^. Thus, the current SH level may correspond mainly to the total glutathione level; however, that requires clarification. Previous studies have reported that thioredoxin system dysregulation may be a factor in the pathogenesis of glaucoma^9^,^10^,^34^,^35^,^36^. The thioredoxin level should be measured separately from other thiol groups.

The results of the current comprehensive large-scale study suggested that lower systemic antioxidant capacity measured by ferric-reducing activity is associated with more severe visual field damage in OAG, which is partly explained by its roles in IOP elevation.

## Methods

### Subjects

The study adhered to the tenets of the Declaration of Helsinki. The institutional review boards of Shimane University Hospital and Iinan Hospital, Shimane, Japan, reviewed and approved the research. All subjects provided written informed consent. In the current study, 202 Japanese subjects with OAG (POAG or NTG) were chosen from a previously established dataset of 531 subjects^12^,^16^. Briefly, OAG was diagnosed based on open iridocorneal angles bilaterally, the characteristic appearance of glaucomatous optic neuropathy such as enlargement of the optic disc cup or focal thinning of the neuroretinal rim, corresponding visual field defects identified using the Humphrey Visual Field Analyzer Swedish Interactive Thresholding Algorithm central 30-2 program (Carl Zeiss Meditec, Dublin, CA) in at least one eye, and no evidence of secondary glaucoma bilaterally. Subjects with the history of untreated IOP of 21 mmHg or higher in at least one eye were considered to have POAG, and other subjects with no history of untreated IOP of 21 mmHg or higher were considered to have NTG.

### Recording clinical parameters and collecting blood samples

To avoid the possible confounding effect of systemic diseases^37^,^38^,^39^, we questioned subjects about a history of severe systemic diseases during an interview before study entry as previously described^12^,^16^. In addition to a history of severe systemic diseases, to adjust for the possible confounding effects of other factors such as differences in nutrition, blood pressure, blood glucose, and smoking habits^40^,^41^,^42^, we recorded the presence or absence of diabetes, current smoking, time since the last meal, and systolic and diastolic blood pressures (SBP and DBP, respectively) and pulse rate before blood samples were collected as well as the IOP measured and the number of glaucoma medications on the day of sample collection, the highest known IOP previously recorded in the medical charts, and visual field MD values. Venous blood specimens were collected from the antecubital vein into evacuated tubes. During all handling procedures, including transportation from the clinical setting to the laboratory and centrifugation, the temperature was maintained at 4 °C. Serum samples obtained by centrifugation of the collected venous blood were stored at 4 °C until oxidative stress measurements.

### Oxidative stress measurements

All blood analyses were performed using a free-radical analyzer system (FREE Carpe Diem, Wismerll Company Ltd., Tokyo, Japan). Based on the manufacturer’s recommendation, all analyses were performed within 48 hours of venous blood collection to avoid falsely high or low results. To analyze the serum levels of reactive oxygen metabolites, antioxidant capacity, and thiol-antioxidant capacity, the dROM, BAP, and SH tests were performed, respectively. The results of dROM testing were expressed in arbitrary units (U.Carr), one unit of which corresponds to 0.8 mg/L of hydrogen peroxide^40^,^43^; the results of the BAP test were expressed in μmol/L of the reduced ferric ions; the results of the SH test were expressed as μmol/L of the SH groups. A comparison of the measured levels of oxidative stress between the non-glaucoma and glaucoma groups was reported previously^12^.

### Statistical analysis

The data are expressed as the means ± standard deviations and analyzed using JMP statistical software version 11.00 (SAS Institute, Inc., Cary, NC). In the analyses of the subjects with bilateral glaucoma, the eye with the worse (lower) MD between both eyes was the study eye. A possible association with the MD was analyzed using the Spearman’s rank correlation test for continuous data (i.e., age; number of glaucoma medications; highest IOP recorded; IOP on the day of sample collection; SBP; DBP; pulse rate; time since the last meal, and dROM, BAP, and SH tests) and the unpaired t-test for categorical data (i.e., sex, glaucoma type, diabetes, and current smoking status). The associations between the MD and oxidative stress parameters also were analyzed by multiple regression analyses; the parameters associated with the MD by univariate analyses (i.e., Spearman’s rank correlation test or unpaired t-test) were substituted into the multiple regression models together with the oxidative stress parameters. *P* < 0.05 was considered statistically significant.

## Additional Information

**How to cite this article**: Tanito, M. *et al*. Association between systemic oxidative stress and visual field damage in open-angle glaucoma. *Sci. Rep*. **6**, 25792; doi: 10.1038/srep25792 (2016).

## Supplementary Material

Supplementary Information

## Figures and Tables

**Table 1:** Demographic subject data, dROM, BAP, and SH values (n = 202).

Parameters	Mean ± SD or no. (%)	Range or no. (%)
Age (years)	70.6 ± 11.3	69.0–72.1
Sex	Men, 92 (46)	Women, 110 (54)
MD (dB)	−10.5 ± 10.1	−31.48–+2.62
Glaucoma type	NTG, 89 (44)	POAG, 113 (56)
No. glaucoma medications	1.3 ± 1.0	0–3
Highest IOP recorded (mmHg)	21.6 ± 8.2	10–58
IOP on blood sampling day (mmHg)	15.4 ± 5.9	5–52
SBP (mmHg)	139.4 ± 19.9	89–211
DBP (mmHg)	76.4 ± 12.5	47–133
Pulse rate (/minute)	75.1 ± 14.5	42–131
Duration since last meal (hours)	3.8 ± 2.0	1–19
Diabetes	Yes, 46 (23)	No, 156 (77)
Current smoking status	Yes, 23 (11)	No, 179 (89)
dROM test (U.Carr)	354.6 ± 62.8	204–555
BAP test (μmol/L)	1,955.8 ± 276.7	1,154.6–2857
SH test (μmol/L)	612.9 ± 98.1	328–884

SD, standard deviation; dB, decibels.

**Table 2:** Possible association between MD (dB) and various continuous parameters (n = 202).

Parameters	r	*P* value
Age (years)	0.1142	0.1055
No. glaucoma medications	−0.4479	<0.0001*
Highest IOP recorded (mmHg)	−0.2421	0.0005*
IOP on blood sampling day (mmHg)	−0.0086	0.9039
SBP (mmHg)	−0.0218	0.7579
DBP (mmHg)	−0.1897	0.0068*
Pulse rate (/minute)	−0.1258	0.0745
Duration since last meal (hours)	−0.2279	0.0011*
dROM test (U.Carr)	−0.0121	0.8644
BAP test (μmol/L)	0.1550	0.0276*
SH test (μmol/L)	−0.0380	0.5918

The correlation coefficient (r) and *P* values are calculated by Spearman’s rank correlation test between the MD and other parameters. *P < 0.05.

**Table 3:** Possible association between MD (dB) and various categorical parameters (n = 202).

Parameters	mean ± SD	mean ± SD	*P*value
Sex	Men, −13.1 ± 10.6	Women, −8.4 ± 9.2	0.0018*
Glaucoma type	NTG, −6.7 ± 8.2	POAG, −13.5 ± 10.5	<0.0001*
Diabetes	Yes, −10.4 ± 11.1	No, −10.5 ± 9.9	0.6074
Current smoking status	Yes, −15.6 ± 10.2	No, −9.9 ± 10.0	0.0198*

The *P* values are calculated using the Mann-Whitney U-test between groups. *P < 0.05.

SD, standard deviation; dB, decibels.

**Table 4:** Possible association between MD (dB) and various parameters analyzed by multiple regression model (n = 202).

	Model including dROM (/U.Carr)			Model including BAP (/μmol/L)			Model including SH test (/μmol/L)		
R2	0.277			0.292			0.277		
P value for fit	<0.0001			<0.0001			<0.0001		
	**R**	***P* value**	**standard β**	**R**	***P* value**	**standard β**	**R**	***P* value**	**standard β**
Sex (men = 0, women = 1)	1.69	0.2305	0.08	1.24	0.3740	0.06	1.75	0.2114	0.09
Glaucoma type (NTG = 0, POAG = 1)	−3.11	0.0281*	−0.15	−2.83	0.0447*	−0.14	−3.11	0.0282*	−0.15
No. glaucoma medications	−3.15	<0.0001*	−0.29	−3.07	<0.0001*	−0.03	−3.14	<0.0001*	−0.29
Highest IOP recorded (/mmHg)	−0.15	0.0973	−0.12	−0.14	0.0927	−0.12	−0.15	0.0967	−0.12
DBP (/mmHg)	−0.07	0.2077	−0.08	−0.07	0.1936	−0.08	−0.06	0.2197	−0.08
Duration since last meal (hours)	−0.52	0.1006	−0.11	−0.56	0.0779	−0.01	−52.00	0.1032	−0.10
Current smoking status (no = 0, yes = 1)	−2.26	0.2840	−0.07	−2.52	0.2295	−0.08	−2.22	0.2938	−0.07
Oxidative stress marker†	0.002	0.8556	0.01	0.005	0.0442*	0.13	−0.001	0.8280	−0.01

^*^P < 0.05. ^†^Indicates either the dROM, BAP, or SH test values for respective models.

dB, decibels; R, regression coefficient.
