# Supplementary material for: Association between systemic oxidative stress and visual field damage in open-angle glaucoma
Source: Sci Rep. 2016 May 11;6:25792. doi: 10.1038/srep25792 (PMC4863173; doi:10.1038/srep25792)
Supplement: Supplementary Information [file srep25792-s1.pdf]

Association between systemic oxidative stress and visual field damage in open-angle glaucoma

Masaki Tanito[1,2], Sachiko Kaidzu[1], Yasuyuki Takai[1] & Akihiro Ohira[1]

[1]Department of Ophthalmology, Shimane University Faculty of Medicine, 89-1 Enya, Izumo, Shimane, 693-8501, Japan. [2]Division of Ophthalmology, Matsue Red Cross Hospital, 200 Horo-machi, Matsue, Shimane, 690-8506, Japan.

Table S1 Dataset

Study ID	Age	Sex	Type	Last meal	SBP	DBP	Pulse	Diabetes	Smoking	dROM	BAP	SH	IOP	Meds	Highest IOP	MD
4	72	M	POAG	5	159	91	94	y	y	462	1522	577	19	1	20	−29.1
9	67	F	POAG	2	128	79	58	n	n	431	1737	582	13	1	17	−22.4
11	74	M	POAG	2	174	94	75	n	n	261	1799	626	11	1	18	−13.7
12	79	F	POAG	2	141	65	81	y	n	304	2008	601	20	1	24	−6.01
15	65	M	POAG	4.5	211	133	92	n	n	309	1821	553	42	1	42	−30
18	50	F	POAG	2.5	140	87	73	n	n	274	2122	564	11	0	14	0.87
20	73	F	POAG	2	172	93	94	n	n	474	1724	636	15	1	15	−20.1
22	42	F	POAG	2	102	60	67	n	n	336	1828	630	23	0	23	−8.77
24	61	F	NTG	3.5	121	71	94	n	n	281	1547	708	13	2	14	−13
25	38	F	POAG	4	138	84	83	n	n	290	1904	692	13	2	21	−17.4
27	61	M	POAG	6.5	139	79	64	y	y	381	1928	620	52	3	52	−25
28	87	M	POAG	2	164	81	81	n	n	308	2029	551	16	0	16	−30
29	71	M	POAG	1	105	56	73	y	n	272	1453	681	13	3	34	−30.4
30	82	M	POAG	2.5	167	96	100	n	n	412	1994	724	15	1	28	−24.2
31	60	M	POAG	13	138	92	67	n	n	314	2013	807	19	0	22	−1.7
34	42	F	NTG	4.5	109	71	84	n	n	255	1926	726	13	1	14	0.83
35	70	M	POAG	5	123	73	91	y	y	327	1655	551	16	0	21.6	−23.7
44	70	M	NTG	4.5	125	71	71	y	n	296	1892	675	16	3	16	−20.7
45	73	M	NTG	6	182	99	56	n	n	528	1262	606	16	3	19	−17.8
50	58	M	POAG	5	147	99	85	y	y	282	2038	777	37	3	37	−11.4
58	44	F	POAG	4	105	63	64	n	n	378	2186	686	13	2	22	−22.9
68	72	F	POAG	3	121	71	68	y	n	337	2303	847	12	1	19	−26.8
72	74	F	POAG	6	130	86	57	n	n	331	2075	648	15	2	30	−9.6
75	83	F	NTG	3.5	142	85	85	n	n	309	2189	572	13	1	20	−16.6
76	61	M	POAG	3	148	92	63	y	n	378	1649	396	47	2	50	−30
77	65	M	POAG	3	138	72	81	n	y	387	2024	474	23	1	26	−18.3
78	60	M	POAG	5	140	73	91	y	n	406	1871	476	20	1	29	−8.36
79	58	F	POAG	2	97	61	99	n	n	384	1927	396	18	3	21	−8.88
80	49	M	POAG	3	140	94	87	n	n	278	1556	580	18	3	28	−14.2
81	63	F	POAG	3	182	98	128	n	n	370	2028	556	26	3	39	−5.02
84	61	M	POAG	3	110	64	60	n	y	358	2196	580	26	2	52	−25
86	64	F	NTG	5	137	91	102	n	n	347	2071	589	12	1	13	−10.6
88	74	M	POAG	3	125	64	80	n	n	318	1968	523	15	1	18	−13.3
90	58	M	POAG	4	142	85	68	n	n	386	1972	766	12	3	20	−25.5
91	73	M	POAG	4	191	110	74	n	n	299	2113	727	15	2	30	−14
93	65	F	POAG	2.5	124	80	90	n	n	450	2078	692	17	1	28	−0.36
97	81	M	POAG	6	138	96	72	n	y	359	2150	615	21	1	24	−23.2
98	57	M	POAG	3	131	82	70	n	n	380	1459	606	19	3	25	−10.4
100	78	M	POAG	4	146	81	131	y	n	329	1878	439	22	0	23	−1.73
101	74	F	POAG	2.5	146	58	79	n	n	265	2061	579	15	1	27	−2.66
103	80	F	POAG	2.5	129	77	83	n	n	362	1980	678	15	2	27	−14.6
108	78	M	NTG	8	134	70	108	n	n	258	2354	463	14	0	14	−2.71
110	63	M	POAG	3.5	130	67	63	y	y	318	2130	725	22	2	22	−2.89
111	64	F	NTG	3.5	126	75	82	n	n	402	1852	753	13	0	13	−4.31
112	73	F	POAG	2	145	63	73	n	n	239	2143	629	12	1	14	−18.3
114	58	F	POAG	3	130	88	70	n	n	379	2149	657	16	2	20	−6.89
116	74	F	POAG	2.5	150	78	78	y	n	280	2398	633	12	0	18	−5.73
117	76	F	POAG	3	138	87	66	n	n	337	2129	613	17	3	17	−28.8
120	54	M	NTG	4.5	137	83	83	n	y	370	1975	665	16	3	17	−11.7
122	54	M	NTG	3.5	137	77	73	n	y	280	1618	642	12	0	17	−7.47
123	76	F	NTG	3	108	60	69	n	n	314	1758	588	18	1	18	−27.7
124	82	F	NTG	5	167	87	85	n	n	440	1775	559	13	0	13	−7.84
126	82	F	POAG	3.5	187	93	114	n	n	282	1660	635	13	1	15	−7.44
128	63	M	POAG	5	136	95	65	n	n	370	1694	618	14	2	20	−19.5
129	68	M	POAG	19	157	92	79	n	n	338	1979	619	12	0	21	−30
131	24	M	POAG	4.5	162	79	88	y	y	330	1608	615	27	0	27	−0.95
132	82	F	POAG	1.5	142	63	94	y	n	356	2121	421	17	1	24	−27.4
136	88	M	POAG	2	132	75	56	n	n	432	2318	599	12	2	17	−23.5
137	75	F	NTG	3.5	169	91	87	n	n	412	2242	593	16	1	16	−14.1
138	77	M	POAG	4	122	60	96	y	y	348	2007	628	14	2	29	−30
139	71	F	POAG	5	202	85	68	n	n	353	2146	598	13	2	13	−29.2
140	83	M	POAG	4	153	89	105	n	n	401	1508	328	11	2	35	−15.1
141	57	M	POAG	6	131	81	75	n	n	376	1155	586	14	2	27	−3.95
142	77	M	POAG	4	117	80	84	n	y	435	1912	516	10	0	18	−21.6
147	82	F	NTG	3	107	74	112	n	n	359	2075	500	16	0	43	−25.8
153	65	M	POAG	2	143	83	73	y	y	311	2135	594	23	3	33	−19.7
156	71	M	POAG	5	134	69	68	y	n	322	1803	574	14	3	26	−18

157	77	M	POAG	5	118	78	83	n	n	219	1963	598	13	2	15	−30
158	68	F	POAG	6	120	78	78	n	n	363	2308	646	13	1	14	−2.98
159	69	M	POAG	4.5	152	72	88	n	n	356	2168	676	14	1	21	−15.3
160	89	F	NTG	5.5	122	80	104	n	n	390	1895	537	12	0	20	−29.4
161	25	F	NTG	5	117	63	62	n	n	555	2289	638	15	0	18	−8.94
162	71	M	NTG	5	144	77	72	n	n	329	1299	549	11	2	17	−12.9
166	58	M	NTG	3.5	128	81	73	n	n	243	2142	678	10	1	15	−1.55
167	75	F	POAG	6	163	87	58	y	n	439	1812	567	17	3	29	−17.6
168	58	F	POAG	5.5	89	62	82	n	n	445	1960	542	18	3	21	−8.88
170	68	M	POAG	4	132	68	98	y	y	285	1841	736	16	2	20	−12.3
171	67	M	POAG	4	126	76	80	n	n	387	1631	507	14	1	22	−14.1
176	58	F	POAG	3.5	126	71	79	n	n	325	2131	668	20	3	25	−23.7
177	62	F	NTG	7	121	75	83	n	n	423	2138	706	9	2	14	−19.9
178	57	M	POAG	2.5	120	89	91	y	n	346	1905	733	13	1	23	−6.35
180	60	M	POAG	2.5	153	94	96	n	n	383	1894	608	20	1	29	−11.2
182	81	F	NTG	2.5	175	66	76	n	n	382	2094	707	13	0	18	−2.41
184	71	F	POAG	4	122	77	75	n	n	409	2163	595	10	1	28	−25.8
186	68	M	NTG	8	178	70	68	n	n	282	2396	517	13	2	16	−11.6
188	84	M	POAG	3	128	70	68	n	n	330	1518	615	11	1	20	−26.7
189	74	F	NTG	1.5	146	82	86	n	n	362	1711	757	23	0	23	−3.81
193	73	F	NTG	3	126	62	58	n	n	277	1676	816	13	0	14	1.18
194	59	F	POAG	3.5	156	90	42	n	n	450	1551	760	12	2	16	−31.1
196	59	F	NTG	6	152	80	76	y	n	403	1489	864	12	3	16	−28.3
202	66	F	POAG	2.5	129	70	59	n	n	299	2001	794	17	2	24	−1.81
203	61	F	POAG	2	149	70	78	y	n	366	1786	707	17	1	22	−1.06
207	80	F	NTG	2.5	150	84	84	n	n	377	2103	543	15	0	15	−0.63
208	84	F	NTG	2.5	133	70	66	y	n	491	2425	473	15	1	18	−3.29
210	83	F	POAG	3	116	72	67	n	n	413	2063	525	15	1	20	0.87
213	78	M	NTG	3	140	74	75	y	n	337	1625	458	14	1	16	−0.46
214	67	F	POAG	3	130	63	66	n	n	315	1945	538	20	1	24	−1.51
215	73	F	POAG	3	179	80	53	y	n	319	2100	417	23	2	27	−4.1
217	83	M	NTG	1.5	124	47	101	y	n	485	1885	628	16	0	21	−3.85
221	72	M	POAG	5	152	99	79	y	n	320	1959	731	14	3	19	−2.88
222	71	M	NTG	3.5	147	88	100	n	n	266	1843	741	11	1	16	−6.39
223	76	F	NTG	3.5	129	57	81	n	n	366	2286	591	13	1	24.1	−7.75
227	61	F	NTG	3	138	88	64	n	n	453	1747	678	19	1	19	−0.9
231	79	M	NTG	2.5	147	76	59	n	n	380	2286	710	20	2	58	−29.1
234	76	F	NTG	4	145	62	61	n	n	338	2105	680	13	1	17	2.13
235	78	M	NTG	2.5	159	77	72	n	n	392	2505	646	11	1	16	−4.23
236	75	F	NTG	4	158	82	91	n	n	346	2234	726	19	1	19	1.12
239	58	M	NTG	4	150	88	62	n	n	282	1939	884	7	2	20	−15.5
242	72	F	POAG	2	144	69	80	n	n	308	2078	567	23	1	25	−4.1
246	77	F	NTG	2.5	159	48	79	n	n	464	2120	588	17	2	22	−8.16
248	68	F	POAG	3.5	140	75	67	n	n	473	2042	577	10	1	21	1.67
251	74	M	NTG	2	105	59	55	y	n	403	1944	331	14	0	14	0.57
252	79	F	NTG	2.5	148	77	58	n	n	380	1909	628	16	1	17	0.01
256	61	M	POAG	3	132	52	52	n	n	399	1685	598	13	2	40	−8.14
258	69	M	NTG	5.5	155	93	70	n	n	304	1297	820	12	2	18	−25.6
262	82	F	NTG	3	140	75	83	n	n	361	2080	544	12	1	19	−2.36
263	77	F	NTG	1.5	132	81	74	n	n	318	2121	551	12	2	17	−14.9
264	69	F	POAG	2.5	120	61	68	n	n	328	1985	660	15	2	27	−2.89
267	85	F	NTG	3	158	76	58	n	n	350	1925	527	16	1	20	−3.43
269	67	M	NTG	2.5	176	93	80	y	y	343	1945	513	10	0	15	2.62
270	85	F	POAG	3	132	65	62	n	n	415	1709	578	15	1	25	−5.24
276	83	F	NTG	3	154	81	67	n	n	313	1916	590	15	1	18	−0.79
287	78	F	NTG	4	141	80	79	n	n	365	2040	542	15	0	16	−2.68
289	88	M	NTG	3.5	123	69	73	y	n	260	2071	470	8	1	12	−7.68
291	53	M	POAG	12	126	78	69	y	y	488	2194	671	26	3	36	−11.2
292	79	M	POAG	5	120	68	91	y	n	346	2003	589	22	1	23	−1.73
298	78	F	POAG	4	132	65	61	n	n	368	1821	554	11	1	20	−13.5
299	73	F	NTG	3.5	135	57	54	y	n	430	1919	564	14	0	19	−0.3
301	77	F	NTG	3	105	67	70	y	n	303	1733	490	14	1	17	−0.34
302	55	M	NTG	4	123	80	84	n	n	435	2038	677	14	1	19	−8.48
307	84	F	POAG	2.5	158	77	82	n	n	270	2143	624	25	1	25	−5.65
308	77	M	POAG	4.5	116	79	82	n	y	448	2017	792	9	0	18	−21.6
310	62	M	POAG	3.5	140	79	67	n	n	328	2094	753	17	3	20	−2.06
311	60	M	POAG	4.5	130	87	85	y	n	258	1959	629	24	3	42	−30.5
312	83	F	POAG	3	114	51	79	n	n	305	1682	786	15	1	19	−5.34
313	83	F	POAG	3	134	81	87	n	n	522	1975	812	12	1	19	−3.55
314	81	M	NTG	2	146	58	51	n	n	311	2514	693	11	1	17	0.06
318	64	M	NTG	2.5	138	76	75	n	n	251	2339	697	11	1	16	−3.05

320	77	F	POAG	3	128	70	74	n	n	380	2163	738	11	1	21	−0.17
330	53	F	POAG	3	155	96	76	n	n	438	2040	806	5	0	56	−7.53
331	80	M	POAG	2.5	132	62	71	y	n	365	1511	693	13	2	18	−17.3
332	57	M	POAG	4	154	78	61	n	n	313	1968	748	13	3	23	−31.5
335	61	F	NTG	3	111	69	73	n	n	354	1892	734	13	0	15	−1.14
349	84	F	NTG	2	124	63	76	n	n	306	2021	662	11	0	15	−3.11
350	85	M	POAG	2.5	119	57	72	n	n	421	1925	521	15	1	27	0.78
358	83	F	POAG	2	121	65	88	n	n	368	1709	572	13	1	42	−23
361	80	F	POAG	2	136	60	88	y	n	313	1864	633	20	1	25	−0.38
363	83	F	POAG	4.5	154	82	64	n	n	465	2017	496	16	1	24	1.9
364	80	M	POAG	2	168	72	58	n	n	400	1517	430	15	1	36	0.12
367	80	F	POAG	2.5	149	73	59	n	n	332	2723	652	11	2	14	−5.64
369	70	F	NTG	3.5	135	75	58	n	n	360	2857	646	11	1	19	−2.91
373	79	F	NTG	4	151	89	114	n	n	436	2056	565	13	1	17	−1.93
375	76	F	NTG	3.5	134	83	55	n	n	271	2162	607	11	1	18	−0.52
378	78	M	NTG	5	143	83	69	n	n	326	2359	366	15	1	17	−0.45
380	54	M	NTG	6	106	60	65	n	y	303	1655	750	15	3	18	−28.9
383	80	M	NTG	2	150	65	62	n	n	204	1833	660	9	0	11	−1.39
389	66	F	NTG	2.5	111	60	78	n	n	230	2071	680	13	0	16	−2.06
397	75	F	NTG	3.5	129	63	59	n	n	374	1568	485	16	0	17	−2.17
399	82	F	NTG	3.5	134	71	78	n	y	262	2197	523	13	1	19	0.68
400	68	M	NTG	4	127	72	57	n	n	334	1967	534	12	2	18	−4.46
401	75	F	NTG	3.5	172	85	72	n	n	379	1242	674	11	1	17	−7.38
402	79	F	NTG	5.5	158	70	64	n	n	387	2043	516	11	1	17	−13.9
404	79	F	NTG	5.5	151	91	81	n	n	408	1955	599	13	1	14	−12.1
405	49	F	POAG	4	160	102	89	n	n	314	2013	663	18	1	25	−18.1
406	85	M	POAG	3	119	60	63	n	n	286	1414	499	11	1	20	−26.7
407	81	M	NTG	5	142	82	77	n	n	312	2021	574	16	3	16	−7.44
414	71	F	NTG	3.5	90	53	62	n	n	346	2123	551	12	0	12	−0.71
416	71	F	NTG	4.5	121	64	55	n	n	340	1792	587	12	1	17	−6.67
420	75	F	NTG	3	162	80	58	y	n	322	2009	623	12	1	14	−5.1
425	80	F	NTG	3.5	181	66	66	n	n	309	1911	558	14	1	16	−1.83
430	63	M	NTG	3.5	180	100	78	y	n	453	2213	605	13	0	13	−0.08
431	55	F	POAG	3.5	123	80	72	n	n	461	2057	624	12	1	22	−12.3
432	70	F	POAG	4	145	80	71	n	n	358	2032	609	21	0	21	−0.53
433	81	M	POAG	4	159	67	59	n	n	343	1321	611	12	2	21	−24.7
437	71	M	NTG	2.5	133	71	88	y	n	300	1978	575	16	0	17	−2.77
439	81	M	NTG	2.5	134	73	73	y	n	335	2224	600	17	2	21	0.52
441	84	F	NTG	3.5	167	97	79	n	n	385	2229	562	10	1	16	−5.1
443	82	M	NTG	3.5	130	65	58	n	n	393	2114	708	12	0	14	−0.22
445	87	M	NTG	4	146	55	55	n	n	380	1473	619	17	0	17	−0.61
450	67	M	POAG	3.5	123	78	77	y	n	393	2048	764	17	1	27	0.23
452	78	F	POAG	3	132	81	78	y	n	416	1960	570	17	1	23	0.86
460	73	F	NTG	3	142	72	68	n	n	413	1943	545	11	0	13	−0.8
464	65	F	POAG	3.5	165	83	52	n	n	469	2259	612	19	1	27	−2.71
469	83	M	NTG	3	162	70	51	n	n	374	2031	580	11	1	17	−2.35
476	67	F	POAG	4.5	137	69	66	n	n	369	1540	523	16	1	25	−7.7
477	73	M	NTG	4	125	64	71	n	n	325	1999	509	13	2	18	−9.76
478	73	F	POAG	2	139	83	80	n	n	341	1742	591	17	0	22	−1.66
484	62	F	NTG	6	124	64	80	n	n	305	2140	646	14	0	14	−5.01
493	64	M	NTG	3	147	89	61	n	y	351	2528	657	13	2	13	−15.9
498	56	M	POAG	2.5	133	90	86	n	y	420	1992	550	15	2	26	−19.8
501	77	F	NTG	3.5	153	70	54	n	n	362	2144	548	6	1	10	−7.51
502	57	M	NTG	4.5	144	81	53	n	y	353	1571	602	14	1	16	−1.44
505	48	F	POAG	3.5	147	98	89	n	n	296	2153	618	19	1	23	−2.83
506	84	M	NTG	5	140	76	66	n	n	397	2665	526	11	0	13	−1.34
507	79	F	NTG	3	149	70	74	n	n	267	2030	630	11	1	12	−2.65
510	61	F	NTG	2.5	120	71	76	n	n	298	1651	616	18	0	18	−0.74
516	71	F	NTG	3	136	70	88	y	n	354	2301	644	13	1	23	1.08
518	76	M	POAG	15	139	71	75	y	n	315	2316	607	14	1	26	−10.4
521	75	F	POAG	4	128	89	67	n	n	402	1972	526	34	3	40	−29.2
526	74	F	POAG	5	146	73	63	n	n	387	1693	496	11	2	18	−7.46
528	76	M	POAG	6.5	144	79	73	n	n	352	1343	538	13	3	25	−29.1
531	82	F	POAG	6.5	135	83	89	n	n	281	2141	489	13	1	21	−24.1
